# A smooth and differentiable bulk-solvent model for macromolecular diffraction

**DOI:** 10.1107/S0907444910031045

**Published:** 2010-08-13

**Authors:** T. D. Fenn, M. J. Schnieders, A. T. Brunger

**Affiliations:** a Department of Molecular and Cellular Physiology and Howard Hughes Medical Institute, Stanford, California, USA; b Department of Chemistry, Stanford, California, USA; c Departments of Neurology and Neurological Sciences, Structural Biology and Photon Science, Stanford, California, USA

**Keywords:** bulk-solvent models

## Abstract

Inclusion of low-resolution data in macromolecular crystallo­graphy requires a model for the bulk solvent. Previous methods have used a binary mask to accomplish this, which has proven to be very effective, but the mask is discontinuous at the solute–solvent boundary (*i.e.* the mask value jumps from zero to one) and is not differentiable with respect to atomic parameters. Here, two algorithms are introduced for com­puting bulk-solvent models using either a polynomial switch or a smoothly thresholded product of Gaussians, and both models are shown to be efficient and differentiable with respect to atomic coordinates. These alternative bulk-solvent models offer algorithmic improvements, while showing similar agreement of the model with the observed amplitudes relative to the binary model as monitored using *R*, *R*_free_ and differences between experimental and model phases. As with the standard solvent models, the alternative models improve the agreement primarily with lower resolution (>6 Å) data *versus* no bulk solvent. The models are easily implemented into crystallographic software packages and can be used as a general method for bulk-solvent correction in macromolecular crystallography.

## Abstract 2

A new method for modeling the bulk solvent in macromolecular diffraction data based on Babinet’s principle is presented. The proposed models offer the advantage of differentiability with respect to atomic coordinates.

## 1. Introduction

The size, shape and crystallographic packing of macromolecules leads to interstitial spaces that occupy a significant portion (typically >40%) of the crystal volume (Matthews, 1968 ▶). The solvent surrounding the protein is typically only visibly ordered within the first shell of hydration, while the scattering of the remainder can be approximated as arising from a continuum. In macromolecular crystallography, this effect is usually modeled by defining a region of zero density inside the solvent-accessible surface, while the area outside is treated as a constant (*i.e.* flat) scattering volume (which we refer to as the ‘binary’ model). The resulting binary mask is Fourier-transformed and added to the atomic structure factors, yielding a total scattering factor **F**_t_, 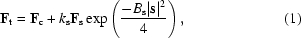
where **F**_c_ are the structure factors computed from the molecule, **F**_s_ are the structure factors from the Fourier-transformed binary mask, *k*_s_ is the electron density of the bulk solvent in units of electrons per Å^3^, *B*_s_ is a *B* factor that represents the isotropic thermal disorder of the solvent and **s** is the reciprocal-lattice vector. The effect of exponential multiplication by *B*_s_ in reciprocal space is smoothing of the bulk-solvent model in real space (Fokine & Urzhumtsev, 2002 ▶).

**Figure 1:**
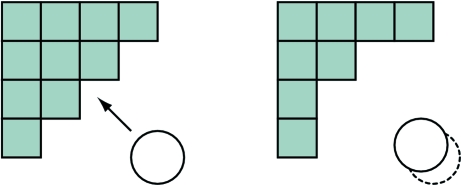
A binary mask (gray squares) can be affected by moving a single atom near the solvent–protein boundary (circle) along the shown vectorial path by an infinitesmally small step, leading to noncontinuous changes in the mask.

The binary-mask model was initially proposed by Phillips (1980 ▶) and later adapted in *X-PLOR* (Jiang & Brünger, 1994 ▶) using a version of the Lee and Richards solvent-accessible surface model (Lee & Richards, 1971 ▶). The constant *k*_s_ and *B* factor *B*_s_ can be optimized against the diffraction data and recent efforts have improved the robustness of the solvent-parameter optimization in *PHENIX* and *CNS* (Fokine & Urzhumtsev, 2002 ▶; Afonine *et al.*, 2005 ▶; Brunger, 2007 ▶; Adams *et al.*, 2010 ▶). This approach represents the current standard and has been incorporated into most modern crystallographic software packages. However, by virtue of the binary nature of the mask used to calculate **F**_s_, this bulk-solvent model is ‘jump-discontinuous’ at the solute–solvent boundary (*i.e.* the mask jumps from a value of zero to one) and therefore is not differentiable with respect to the atomic coordinates. As a result, chain-rule terms arising from the binary mask cannot be included during optimization of the coordinates using positional minimization or simulated annealing (Brünger *et al.*, 1987 ▶). Therefore, the bulk-solvent model is kept fixed until an update is performed; thus, the overall target function is not continuous. A potential problem with the binary mask is made apparent by considering that values in the mask can be flipped upon infinitesimal atomic coordinate changes (see Fig. 1 ▶).

The application of Babinet’s principle to macromolecular crystallography was originally proposed by Moews & Kretsinger (1975 ▶) and involves using the same Fourier coefficients as derived from the atoms (**F**_c_ in equation 1) but with opposite phases to describe the bulk-solvent scattering. An overlooked aspect of this approach is that the bulk solvent is differentiable with respect to the individual atoms and relies on the same derivatives as computed for the atomic model structure factors. However, the use of Babinet’s principle as a bulk-solvent model is uncommon owing to the poor agreement with the diffraction data relative to the binary model. This is a consequence of the fact that the phase-inverted **F**_c_ is not an adequate description of the electron density in the bulk-solvent region, as it is not a characteristic function that shows the relatively featureless electron density characteristic of bulk solvent. Here, we propose a modification of Babinet’s principle that uses a characteristic function rather than the **F**_c_s and retains differentiability with respect to the atomic co­ordinates.

To generate a differentiable characteristic function, we first use atom-centered Gaussians, which has been suggested and implemented in various contexts in the past. Phillips’ initial description of a bulk-solvent correction used Gaussians, but the resulting Gaussian density was isocontoured at a selected density level where all points outside the isocontour were set to a constant density and all points inside were set to zero, yielding a binary model (Phillips, 1980 ▶). Roversi and coworkers used Gaussian smoothing of the molecular surface to assist *ab initio* phasing methods (Roversi *et al.*, 2000 ▶). Kostrewa suggested the use of exponential smoothing as an improvement over the binary model for crystallographic data sets (Kostrewa, 1997 ▶; Fokine & Urzhumtsev, 2002 ▶), and a Gaussian model for permittivity and ionic strength is common in biomolecular Poisson–Boltzmann calculations (Grant *et al.*, 2001 ▶).

As an alternative to atom-centered Gaussians, we use a polynomial switch at the solute–solvent boundary. The simplicity of low-order polynomials offers a potential speed benefit over the Gaussian treatment, which is an important consideration for macromolecules with many atoms. The utility of polynomials and their derivatives for describing solute–solvent boundaries has been duly noted (Im *et al.*, 1998 ▶; Schnieders *et al.*, 2007 ▶) and is a critical part of Poisson–Boltzmann calculations for large systems (Baker *et al.*, 2001 ▶). Polynomials have also been noted to stabilize molecular-dynamics simulations in which implicit bulk-solvent models are used (Arnold & Ornstein, 1994 ▶).

We describe a simple replacement of the solvent-model structure factors **F**_s_ using either a polynomial switch (which we refer to as the ‘polynomial’ model) or a smoothly thresholded version of atom-centered Gaussians (referred to as the ‘Gaussian’ model). We show that the polynomial and Gaussian models result in continuous target values as a function of coordinates and similar agreement with the diffraction data as the binary model, as monitored using *R*, *R*_free_ and differences between model and experimental phases. Finally, the Gaussian and polynomial models are differentiable with respect to atomic coordinates such that chain-rule terms arising from the bulk-solvent model can be included during positional minimization and simulated-annealing protocols.

## 2. Methods

### 2.1. Babinet’s principle

**Figure 2:**
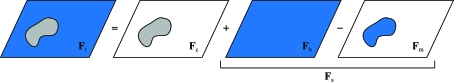
The total scattering of a macromolecule (gray) in bulk solution (blue) can be described as the sum of the scattering from the macromolecule alone (**F**_c_) plus constant bulk scattering (**F**_b_) minus the solute mask (**F**_m_).

The total scattering of a macromolecule in bulk solution is depicted pictorially in Fig. 2 ▶. The scattering from the macromolecule alone (gray; **F**_c_) is added to the constant scattering from a bulk scattering mass (blue; **F**_b_) minus the bulk scattering effect that would arise from the macromolecular mask alone (**F**_m_). For the sake of simplicity, the symmetry of the system is assumed to be *P*1. The situation simplifies in reciprocal space, as the Fourier transform of the constant scattering volume is zero (except at zero frequency, which is ignored in this case). Therefore, (1) can be reformulated as
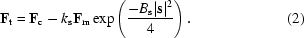
This is Babinet’s principle as it is typically applied in macromolecular crystallography, with the exception of inverting the phase of **F**_m_ rather than **F**_c_. This is therefore opposite to the binary model in that the real-space mask is one inside the protein mask and zero elsewhere. **F**_m_ can be any function that varies from zero in the bulk solvent to one in the solute region.

For both the polynomial and Gaussian models presented below, we assume *n* atoms at individual coordinates **r**_*i*_ and an arbitrary grid point at **r**_*g*_. The distance between the two vectors is defined as *r* = ||**r**_*g*_ − **r**_*i*_||.

### 2.2. Gaussian model

The derivation of the Gaussian bulk-solvent model follows that of Grant *et al.* (2001 ▶). Briefly, starting with a Gaussian with variance σ^2^, 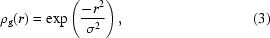
a function for the bulk mask at grid point **r**_*g*_ can be defined as a product of the densities of individual atoms, 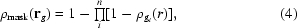
which, ignoring atomic overlaps, is approximately equivalent to
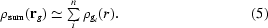
To generate a characteristic function that smoothly varies from zero to one, the solute mask (note that the general term ‘mask’ can be any density function, not just limited to values of zero and one as for a binary mask) is
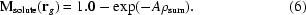

               *A* is a constant that scales the Gaussians. For this work, we chose a value of 11.5 Å based on the results of Grant *et al.* (2001 ▶).

Computation of the solute mask requires two loops, the first being over the atoms (to generate ρ_sum_) and the second over the map to carry out the exponentiation in the above equation. This is similar to the binary bulk-solvent model, which requires an initial pass through the atoms to generate the mask and a second pass to shrink the mask based on the shrink radius (Jiang & Brünger, 1994 ▶).

This mask can be Fourier-transformed to yield **F**_m_ in (2). The solvent mask, which is necessary for the derivatives (see equation 9), is simply 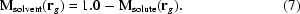
The Gaussian functions provide an easily differentiable formalism with respect to the atomic coordinates,
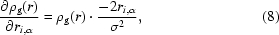
where α ∈ {*x*, *y*, *z*}. This equation can be combined using the chain rule to yield the derivative of the bulk solvent at **r**_*g*_ for atom *i*,
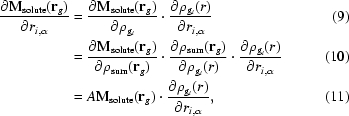
which can be used with any target-function derivative with respect to the bulk-solvent structure factors following the equations given in Brünger (1989 ▶).

It is worthwhile to point out that the atomic Gaussians used in the computation of **F**_c_ could be used for the purposes of generating the solute/solvent mask. However, this would come at a significant computational expense as the calculations for the Gaussian and polynomial model are performed in *P*1 (see *Implementation* section[Sec sec2.4]).

### 2.3. Polynomial switch model

For the polynomial model, we implemented a multiplicative cubic switch function with the endpoints fixed at zero and one (Im *et al.*, 1998 ▶), although higher order functions are also possible (Schnieders *et al.*, 2007 ▶). Given an atom radius *a* and a window size to compute the switch function *w*, the distance *d* between the grid point and atom is computed as *r* − *a* + *w*. The cubic polynomial function describing the solvent density is then 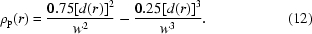
The switching function *S* is only computed within the window *w*, 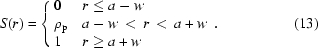
The characteristic function to yield the solute mask is the product of the switch functions over atoms,
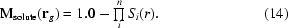
This has the benefit that only a single pass through the atoms is required, which provides a speed benefit over the Gaussian model (data not shown). As above, the solute mask can be used to compute **F**_m_ and the bulk-solvent density is simply
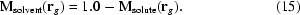
The derivative of the switch with respect to atomic coordinates is only necessary inside the window region:
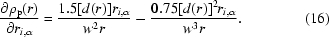
The derivative of the solute mask with respect to atomic co­ordinates can be obtained by
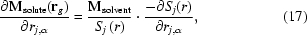
which, as with the Gaussian model, can be combined using the chain rule with the refinement target function of choice.

### 2.4. Implementation

An important point regarding the implementation of the described models is the presence of the real-space solvent mask in the derivatives (equations 9 and 17). This requires that the solvent mask is stored in memory. Furthermore, as the solvent mask is a many-body equation (*i.e.* the solvent density at any given grid point may depend on several atoms, including those generated by crystallographic symmetry), the solvent mask must include contributions from the unique atom set and nearby symmetry atoms. To properly account for this, we first use the solute mask in computing **F**_m_ (equations 6 and 14) and then use a spatial decomposition routine to locate atoms within a 4.0 Å shell around the unique atom set, although the specific choice of this shell depends on the parameters of the bulk-solvent model (*e.g. w* in the polynomial model). This calculation is performed in *P*1. The resulting mask is converted to a solvent mask (*e.g.* equation 7) and used for the derivative computation.

The Gaussian and polynomial models were computed with an experimental program system, *Force Field X* (*FFX*), which is a Java Virtual Machine (JVM) based framework aimed towards combining modules from several fields of molecular biophysics into an integrated platform (Schnieders & Fenn, in preparation). Bulk-solvent structure factors from *FFX* were input to *CNS* v.1.3 (Schröder *et al.*, 2010 ▶; Table 1 ▶). The *CNS* optimization of the solvent parameters used a grid search together with least-squares optimization (Brunger, 2007 ▶). The least-squares optimization employed a least-squares target function [see equation 2 in Brünger (1989 ▶) and equation 11 of Jiang & Brünger (1994 ▶)]. The bulk-solvent models were computed on a bounded grid that was calculated as one-third of the maximum resolution, but limited to 0.57 and 0.9 Å for high- and low-resolution structures, respectively (Rees *et al.*, 2005 ▶; Brunger, 2007 ▶). The default probe and shrink radius parameters (1.0 Å) were used for the binary model in *CNS*.

We also optimized the solvent models within the *FFX* framework (Supplementary Table 1 ▶ 1). These optimizations started from a scale (*k*_s_) that represents the electron density of bulk water at 267 K and standard pressure, 0.33 e Å^−3^ (Botti *et al.*, 2002 ▶), and a *B* factor (*B*_s_) of 50.0 Å^2^ (Fokine & Urzhumtsev, 2002 ▶). *FFX* employed the derivatives given in the appendix of Afonine *et al.* (2005 ▶) for the optimization of *k*_s_ and *B*_s_. Analytic gradients were verified using finite-difference methods and optimizations were carried out using a limited-memory BFGS minimizer until the r.m.s. gradient magnitude was reduced to less than 1.0 × 10^−5^. In contrast to *CNS*, combined solvent-parameter grid searches/minimizations were not performed. Furthermore, the solvent-model grid size was simply computed as one-third of the maximum resolution (Bricogne, 2006 ▶), *i.e.* no grid bounding was performed.

## 3. Results

For the test cases used here, only the anisotropic scale and bulk-solvent parameters were fitted to the diffraction data. Atomic coordinates were not altered from their deposited values and no refinement of the positions or atomic *B* factors was performed (except for testing of the analytic derivatives; see below). All atoms, including ordered water molecules, were included as part of the molecular surface for use in computation of the solvent mask (*e.g.* equations 6 and 14). However, TLS-based (Schomaker & Trueblood, 1968 ▶) ANISOU records from the PDB files and ligands were ignored (MPD in the case of PDB entry 1n7s, zinc and phosphate in PDB entry 3dyc, GTP in PDB entry 3bbp and ATP in PDB entry 1nsf) owing to a lack of support for these ligands in the software used.

**Figure 3:**
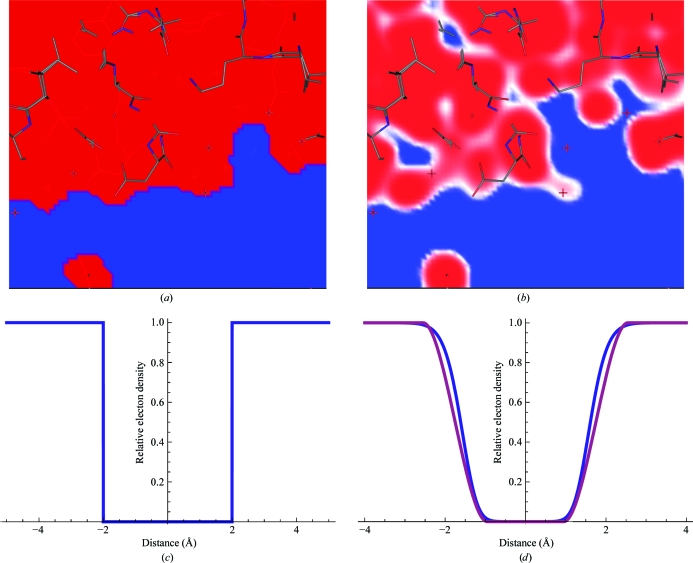
Density slices from (*a*) a binary model mask and (*b*) the corresponding Gaussian model mask (the polynomial model mask is similar and is not shown) derived from test model 1exr. The coloring scheme is from red (zero density) through white (0.5 density level) to blue (1.0 density level). Below each density slice is a one-dimensional representation of the mask for each case, given a single atom at the origin. The distance from the atom in Å is given on the *x* axis and the *y* axis depicts the relative electron density of the mask. In (*d*), the Gaussian model (*A* = 11.5 Å, σ = 0.55 times the van der Waals radius) is shown in blue and the polynomial model (*a* set to the van der Waals radius, *w* = 0.8 Å) is shown in magenta given an atom with a van der Waals radius of 1.75 Å.

We initially sought to determine optimum values for the two variable parameters of the alternative bulk-solvent models: σ for the Gaussian model and 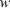 for the polynomial model (the value of *a* in the polynomial model was set to the van der Waals radius). Using a grid search with the *R* and *R*_free_ values as a guide, a value of 0.55 times the van der Waals radius was determined for σ to consistently yield optimum *R* values (data not shown). In some cases, the optimum value for σ varied slightly; it is straightforward for these cases to implement grid-search routines as part of the refinement process (Brunger, 2007 ▶). For the polynomial model, we determined a value of 0.8 Å for *w*, which yielded similar *R* values to the Gaussian model. Furthermore, the profiles of the polynomial and Gaussian models with these values appeared to be similar (Fig. 3 ▶ *d* and discussion below), suggesting that the two masks model the bulk solvent in a similar fashion.

A density slice of the binary and Gaussian bulk-solvent models is presented in Fig. 3 ▶. Of note is the continuous smooth transition from the protein region (red) to bulk solution (blue) in the Gaussian/polynomial mask (Fig. 3 ▶ *b*) *versus* the sharper transitions in the binary mask (Fig. 3 ▶ *a*). The other apparent feature of the density slices is that the alternative bulk-solvent models do not use a probe radius to extend the solvent mask and therefore the resultant masks are more closely associated with the van der Waals surface definition of Richards (1977 ▶) and Connolly (1985 ▶) rather than the solvent-accessible surface that defines the binary model (Lee & Richards, 1971 ▶). The shrink procedure of the binary mask reduces the solvent-accessible surface to the molecular surface, with the intention that small internal cavities are excluded from the mask (compare Figs. 3 ▶ *a* and 3 ▶ *b*). This has the effect of preventing bulk-solvent scattering in regions that should not scatter X-­rays, such as hydrophobic cavities. However, it is not determined from the mask procedure what type of contacts are available (if any) in the excluded cavities to differentiate a cavity as hydrophobic or otherwise (Finney, 1975 ▶). In any case, significant experimental evidence suggests that internal cavities – even small hydrophobic cavities – can be partly occupied by water molecules that exhibit short bound lifetimes or are dynamically disordered such that the water molecules are not observed in crystallographic experiments (Richards, 1977 ▶; Tilton *et al.*, 1986 ▶; Ernst *et al.*, 1995 ▶; Buckle *et al.*, 1996 ▶; Otting *et al.*, 1997 ▶; Yu *et al.*, 1999 ▶; Liu *et al.*, 2008 ▶). Therefore, the use of the van der Waals surface in the alternative bulk-solvent models has some physical footing, although it is not entirely correct since the average electron density in these small internal cavities is expected to be less than that of the bulk solvent. Nevertheless, we obtain similar *R* values with the binary, Gaussian and polynomial models (see discussion below) and the difference densities in small cavities appear to be similar (Supplementary Fig. 1 ▶ 1). A pictorial representation of the mask is also shown in the one-dimensional case for an atom located at the origin below each two-dimensional density slice using either the binary model (Fig. 3 ▶ *c*) or the Gaussian/polynomial model (Fig. 3 ▶ *d*). The Gaussian and polynomial models generate a solvent distribution that asymptotes to bulk electron density approximately where the first shell of density in radial distributions of solvent about protein molecules appears (Pettitt *et al.*, 1998 ▶; Makarov *et al.*, 2002 ▶; Chen *et al.*, 2008 ▶).

**Figure 4:**
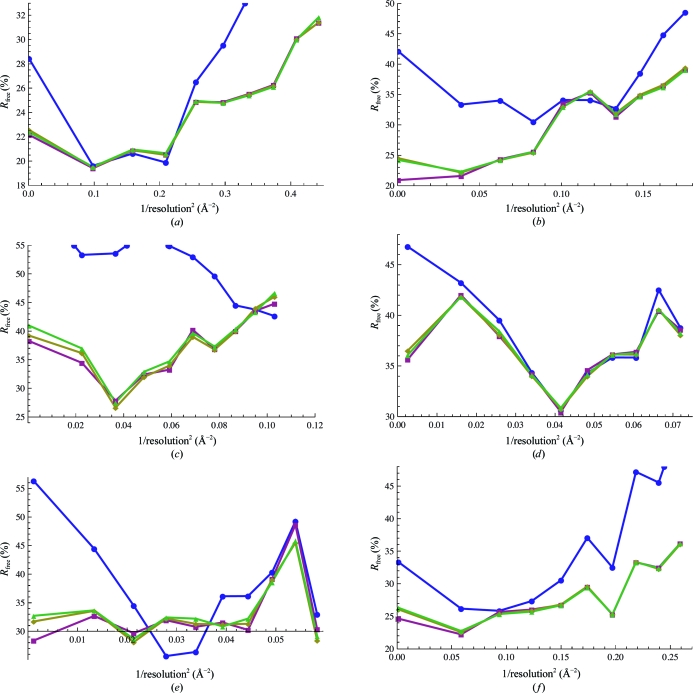
*R*_free_ values as a function of resolution. Blue circles correspond to no bulk-solvent correction, magenta squares to the binary model, yellow diamonds to the polynomial model and green triangles to the Gaussian model. Only a single overall scale factor was used for the *R*-value calculations rather than a resolution-dependent scale for the sake of consistency with the overall *R* values. (*a*) 1n7s, (*b*) 3dyc, (*c*) 3bbp, (*d*) 2du7, (*e*) 3bbw, (*f*) 1nsf.

The similar *R*_free_ of the Gaussian and polynomial models compared with the binary model (Table 1 ▶ and Supplementary Table 1 ▶ 1) suggests that the alternative bulk-solvent models are similarly consistent with the diffraction data. This is also reflected in the agreement between the calculated and experimentally determined phases in the 1nsf test case. Fig. 4 ▶ shows that the bulk-solvent models (magenta, yellow and green lines) primarily improve the *R*_free_ at a resolution lower than approximately 6 Å (0.03 Å^−2^) compared with having no bulk-solvent correction (blue lines), although the agreement with the high-resolution data is also improved in most cases. The *k*_s_ values are higher on average for the polynomial/Gaussian models *versus* the binary model, perhaps compensating for an overall smaller solvent electron-density volume owing to the soft nature of the transition at the solvent–solute boundary compared with the binary model.

**Figure 5:**
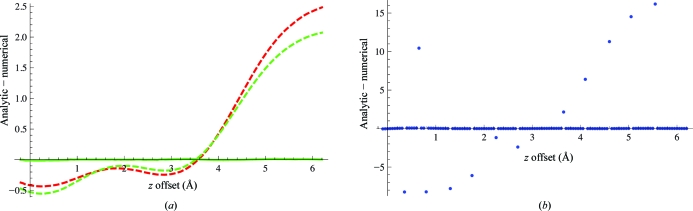
Difference between analytic and numerical derivatives (*y* axis) upon moving a solvent water atom through bulk solution (*x* axis). (*a*) The solid lines show the derivatives and finite differences calculated using either the polynomial model (green) or the Gaussian model (red) and a Δ (18) of 1.0 × 10^−4^ Å. The solid green and red lines overlap and thus only the green line is visible. Dashed lines represent the differences if the derivatives with respect to bulk solvent are not included in the total. (*b*) Derivatives calculated by finite differences using the binary model, with solvent-model updates performed at every step and a Δ of 0.01 Å.

To test the analytic gradients of the alternative bulk-solvent models, a solvent water molecule in one of the tested structures (water 2126 in model 1exr) was moved from its original position in 0.05 Å increments into the bulk solvent while monitoring the analytic atomic derivatives compared with finite differences of the log likelihood [LLK; for details on the computation of the log-likelihood target, see Cowtan (2005 ▶) and McCoy (2004 ▶)]. Finite differences were calculated using a double wide criterion
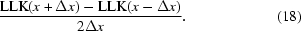
For this procedure, the scale and *B*-factor values of the bulk solvent were held fixed as the water molecule was moved. The results are shown in Fig. 5 ▶. Using a Δ*x* of 1.0 × 10^−4^ Å (Fig. 5 ▶ *a*), the derivatives and finite differences match if derivatives based on either (9) or (17) are included (solid lines), but do not agree (dashed green and red lines in Fig. 5 ▶ *a*) if the derivatives of the bulk solvent are not included.

The finite differences for the binary model with solvent-model updates performed at every step (blue dots in Fig. 5 ▶ *b*) show larger fluctuations than the corresponding calculation without derivatives for the Gaussian and polynomial models (compare the dashed lines in Fig. 5 ▶ *a* and the dotted lines in Fig. 5 ▶ *b*). Δ*x* was set to 0.01 Å in the binary case to avoid aliasing artifacts. Finer grid spacings could not improve this result. This example illustrates that the alternative bulk-solvent models will be less prone to sawtooth-like (*i.e.* up and down) patterns during bulk-solvent model updates (see, for example, Fig. 2 in Phillips, 1980 ▶). However, computation of the solvent model and its derivatives are required at every minimization or simulated-annealing step to achieve the improved stability.

## 4. Conclusions

Implicit continuum solvent models are important for crystallographic refinement to improve the agreement between model and diffraction data at low resolution and such models also have potential for improving phasing methods (Roversi *et al.*, 2000 ▶). The utility of a polynomial or Gaussian definition of the solvent density extends beyond crystallography, as continuum solvent electrostatics are a crucial component in analyses such as computations of binding and desolvation energies (for a review of this subject, see Kollman *et al.*, 2000 ▶), as well as p*K*_a_ calculations, for which accurate continuum models and their derivatives are crucial in improving agreement with experiment (Simonson *et al.*, 2004 ▶). It is also possible to combine implicit models based on reference interaction-site models (Lounnas *et al.*, 1994 ▶) and explicit solvent, although both come with a greater time cost. These methods may be of interest in accounting for differences in solvation between multiple structures and to fully analyze the pattern of hydration around macromolecules (Makarov *et al.*, 2002 ▶); studies of protein structures have suggested the need for such methods for quite some time (Savage & Wlodawer, 1986 ▶). Furthermore, crystal structures obtained with highly accurate experimental phases suggest that the outer shells of solvation about proteins may not be captured by a simple continuum model (Burling *et al.*, 1996 ▶).

The polynomial and Gaussian continuum solvent models offer a comparable agreement with the diffraction data *versus* the standard binary model as the *R* values and phase differences suggest. The continuous nature of the alternative models offer improved stability for atomic refinement, the latter of which acts as a ‘continuum boundary’ on the atoms. These aspects of the polynomial and Gaussian models will be most powerful when the model is updated at each step during the refinement process.

## Supplementary Material

Supplementary material file. DOI: 10.1107/S0907444910031045/mn5003sup1.pdf

## Figures and Tables

**Table 1:** Bulk-solvent statistics for several test structures *k*_s_ is the bulk-solvent scale term, *B*_s_ is the bulk-solvent *B*-factor term and |Δϕ| is the phase difference between the model and an experimentally determined phase set. The binary model uses the standard probe and a shrink radius of 1.0 Å. The *w* value was held fixed at 0.8 Å for the polynomial model and the *A* and σ parameters for the Gaussian model were fixed at 11.5 Å and 0.55 times the van der Waals radius, respectively. ‘None’ refers to the *R* values calculated without a bulk-solvent model. The binary model was generated with *CNS* and the polynomial and Gaussian models were generated with *FFX* and then imported into *CNS*. Solvent-parameter optimization and analysis was carried out with *CNS*.

PDB code	*d*_lim_ (Å)	Model	*k*_s_ (e Å^−3^)	*B*_s_ (Å^2^)	*R* (%)	*R*_free_ (%)	|Δϕ| (°)
1n7s	1.45	None	—	—	23.99	25.66	—
		Binary (*CNS*)	0.42	52.2	20.00	22.17	—
		Polynomial	0.45	61.1	20.14	22.32	—
		Gaussian	0.50	70.3	20.15	22.29	—
3dyc	2.3	None	—	—	30.23	36.27	—
		Binary (*CNS*)	0.34	33.7	20.82	26.54	—
		Polynomial	0.40	61.2	21.42	27.42	—
		Gaussian	0.42	67.0	21.48	27.35	—
3bbp	3.0	None	—	—	39.31	55.24	—
		Binary (*CNS*)	0.32	62.3	31.56	35.50	—
		Polynomial	0.34	62.9	32.29	35.65	—
		Gaussian	0.35	64.5	32.38	36.59	—
2du7	3.6	None	—	—	34.36	39.82	—
		Binary (*CNS*)	0.25	102.3	31.39	36.31	—
		Polynomial	0.25	89.4	31.27	36.50	—
		Gaussian	0.28	104.0	31.33	36.46	—
3bbw	4.0	None	—	—	35.11	40.31	—
		Binary (*CNS*)	0.38	43.3	30.15	32.41	—
		Polynomial	0.35	19.6	29.92	32.83	—
		Gaussian	0.38	34.4	30.37	33.26	—
1nsf	1.9	None	—	—	32.11	31.80	39.86
		Binary (*CNS*)	0.40	62.2	25.66	25.84	38.51
		Polynomial	0.40	71.0	26.07	26.26	38.69
		Gaussian	0.42	91.8	26.13	26.33	38.78

## Footnotes

1 Supplementary material has been deposited in the IUCr electronic archive (Reference: MN5003). Services for accessing this material are described at the back of the journal.
