# Supplementary material for: A smooth and differentiable bulk-solvent model for macromolecular diffraction
Source: Acta Crystallogr D Biol Crystallogr. 2010 Aug 13;66(Pt 9):1024–31. doi: 10.1107/S0907444910031045 (PMC2935282; doi:10.1107/S0907444910031045)

Supplemental Information

Supplemental Table 1: Bulk solvent statistics for several test structures as calculated exclusively in FFX. k_s is the bulk solvent scale term, B_s is the bulk solvent B factor term, and $|\Delta\phi|$ is the phase difference between the model and an experimentally determined phase set. The binary model uses a probe and shrink radius of 1.0 Å. The w value was held fixed at 0.8 Å for the polynomial model, and the A and σ parameters for the Gaussian model were fixed at 11.5 Å and 0.55 times the van der Waals radius, respectively. "None" refers to the R values calculated without a bulk solvent model. The slight differences in R and R_{free} values between the vales presented in Table 1 and this table can be in part attributed to differences between CNS and FFX not related to the solvent model. FFX does not utilize bounded grids for the solvent structure factor calculation (hence we distinguish between the two different binary models as indicated by "CNS" and "FFX"). In CNS, the solvent parameter optimization and the R values are performed for $\mathbf{F_o}$ that have been made pseudo-isotropic by application of the inverse of the anisotropic component of the overall B-factor tensor, whereas FFX uses the original $\mathbf{F_o}$.

PDB ID	d_{lim} (Å)	model	k_s	B_s	R	R_{free}	$\lvert\Delta\phi\rvert$
1N7S	1.45	none	-	-	22.38	24.12	-
		binary (FFX)	0.40	32.8	19.79	22.23	-
		poly	0.53	45.7	19.44	21.54	-
		gauss	0.54	43.4	19.25	21.33	-
3DYC	2.3	none	-	-	25.93	31.52	-
		binary (FFX)	0.37	49.2	22.03	27.53	-
		poly	0.43	38.4	20.33	26.58	-
		gauss	0.46	42.6	20.25	26.45	-
3BBP	3.0	none	-	-	36.77	38.34	-
		binary (FFX)	0.37	86.2	33.05	36.23	-
		poly	0.41	71.2	31.37	35.02	-
		gauss	0.44	78.4	31.37	35.13	-
2DU7	3.6	none	-	-	34.24	39.37	-
		binary (FFX)	0.31	166.2	32.43	37.92	-
		poly	0.36	80.3	30.54	35.77	-
		gauss	0.39	89.5	30.66	35.76	-
3BBW	4.0	none	-	-	35.09	38.67	-
		binary (FFX)	0.38	21.9	30.34	33.24	-
		poly	0.43	17.4	28.59	30.62	-
		gauss	0.46	25.3	28.88	31.08	-
1NSF	1.9	none	-	-	27.78	27.95	40.66
		binary (FFX)	0.42	52.8	25.48	25.60	39.20
		poly	0.45	53.3	25.13	25.38	38.99
		gauss	0.48	62.2	25.20	25.39	38.98

Supplemental Figure 1: Negative difference density in a hydrophobic pocket region of test model 1N7S. The σ_A weighted $F_o - F_c$ difference density is shown in red, contoured in both cases at -2σ. A) map computed with the binary bulk solvent model (using CNS to generate the binary mask) and B) map computed with the polynomial bulk solvent model.

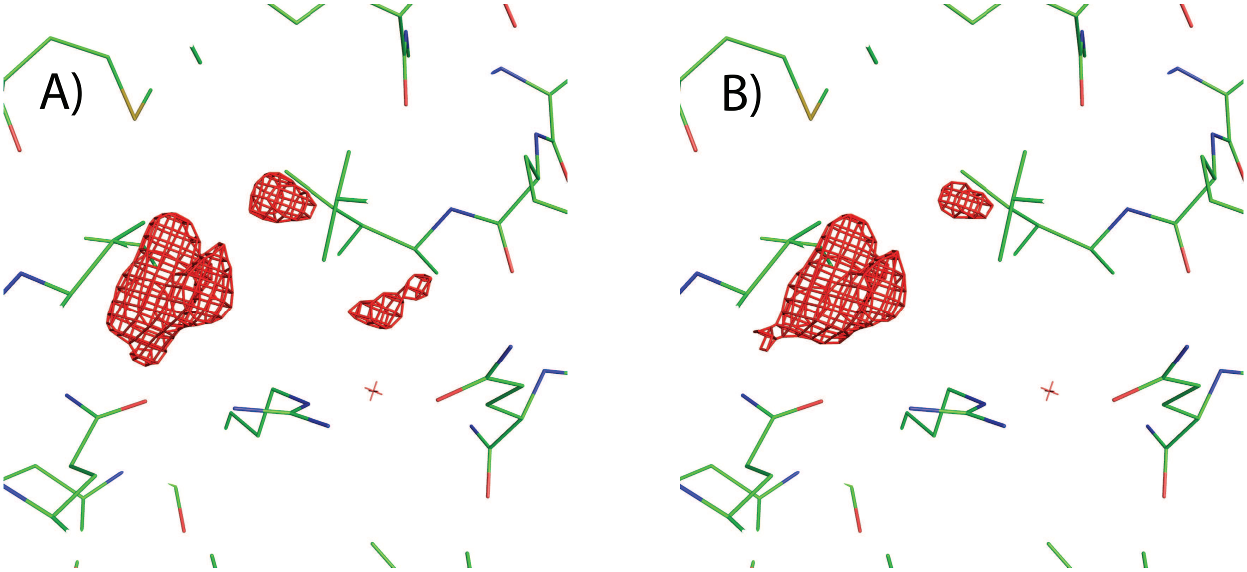

Supplement: Supplementary file 1 [file d-66-01024-sup1.pdf]
